# Orthopedic Injuries Associated With Electric Scooter (E-scooter) Use: Epidemiology, Injury Patterns, Management Strategies, and Prevention

**DOI:** 10.7759/cureus.95141

**Published:** 2025-10-22

**Authors:** Zubair Younis, Muhammad A Hamid, Thomas Devasia, Faliq Abdullah

**Affiliations:** 1 Orthopedics, The Royal Wolverhampton NHS Trust, Wolverhampton, GBR; 2 Orthopedic Surgery, University Hospitals Birmingham NHS Foundation Trust, Birmingham, GBR; 3 Trauma and Orthopedics, The Royal Wolverhampton NHS Trust, Wolverhampton, GBR; 4 Trauma and Orthopedics, University Hospital of Wales, Cardiff, GBR

**Keywords:** electric scooter, e-scooter, fractures, micromobility, orthopedic injuries, trauma

## Abstract

Electric scooters have rapidly become a popular form of micromobility worldwide, offering convenience and affordability. However, their increasing use has been paralleled by a notable rise in emergency presentations, with orthopedic trauma representing a substantial proportion. Injuries often involve young adults, typically following falls, and are characterized by a high incidence of fractures, particularly of the upper limb. Importantly, a significant number of cases require operative management, including complex reconstructions for open or periarticular injuries. The injury spectrum extends beyond simple fractures, with reports of severe lower limb, pelvic, and axial injuries, some resembling those sustained in high-energy trauma. These presentations impose considerable resource demands, reflected in operating theater use, inpatient admissions, and rehabilitation needs. Despite this burden, long-term functional outcome data remain limited, underscoring the need for prospective registries. Management strategies largely align with established trauma principles, but clinicians must maintain a high index of suspicion for multisystem involvement and anticipate challenging reconstructions. Prevention efforts are crucial, with helmet use, speed regulation, intoxication policies, and infrastructure improvements emerging as key modifiable factors. This review synthesizes current evidence on the epidemiology, injury patterns, management, and prevention of e-scooter-related orthopedic trauma.

## Introduction and background

Electric scooters (e-scooters) have rapidly expanded as a micromobility option in cities worldwide, prized for their convenience, low cost, and space efficiency. However, their rapid growth has been accompanied by a sharp and concerning rise in ED visits, with orthopedic trauma representing a significant portion of this burden. Clinical evidence now confirms that a substantial proportion of e-scooter injuries are fractures, many of which require operative management. This underscores that these are not merely minor low-energy falls but often high-energy events that strain orthopedic services [1-3].

Epidemiologic descriptions from international urban centers reveal a consistent injury profile. Studies consistently show that injured riders are typically young adults, the most frequent mechanism is a fall, and upper-limb fractures are common. Perhaps most critically, surgical treatment is required in a sizable fraction of cases, highlighting the clinical severity of these presentations [1-6]. Furthermore, multicenter series document serious injuries, including open fractures, femoral neck fractures in young adults, and multisystem trauma that were previously more associated with high-energy mechanisms, illustrating the significant economic and service burden imposed on trauma systems [5-7].

Key risk factors and modifiers have emerged across studies, including alarmingly low helmet use among riders, a high frequency of incidents during evening and summertime hours, and a nontrivial proportion involving intoxication. The coexistence of privately owned and rental vehicles further complicates the caseload, while differences in local regulation and infrastructure are also recognized as key determinants of incidence and injury patterns [4,5,7].

Given the breadth of these clinical presentations and their potential for serious, function-limiting outcomes, a focused synthesis of orthopedic-specific injury patterns, management pathways, and prevention strategies is urgently needed to inform trauma services and policymakers. This narrative review synthesizes the current orthopedic evidence to quantify the injury burden, outline effective management, and evaluate preventive measures. The ultimate aim is to identify where clinical practice and public health interventions may most effectively reduce harm.

## Review

Epidemiology

The increasing popularity of e-scooters has been mirrored by a sharp rise in emergency presentations worldwide. Since their introduction in 2017, incidence rates of e-scooter-related injuries have escalated in multiple regions. In the United States, Aizpuru et al. reported an increase from 1.53 to 9.22 injuries per 100,000 population between 2014 and 2019 [8]. Similarly, in New Zealand, weekly presentations rose from two to 35 after the launch of an urban scooter-sharing scheme [2]. While injury patterns show global consistency, significant regional variations exist, particularly in helmet compliance and the regulatory environment, which influence incidence and severity.

Demographically, patients are most often young adults. Across European series, the median age of injured riders is consistently in the early thirties [1-3,6]. Coelho et al. described 397 patients in Barcelona with a mean age of 30.8 years, of whom 59.9% were male [1]. Dela Cruz et al. reported similar findings in London, where 83% of riders were male and the median age was 32 years [2]. Hourston et al. observed a mean age of 35.4 years among UK cases [3]. Children and adolescents, however, are not exempt; between 12% and 17% of cases in larger cohorts involved riders under 16 years [1,2].

Across these cohorts, 85-90% of incidents involved the rider falling rather than colliding, and over 60% occurred between 3 p.m. and 11 p.m. [1,2]. Seasonal peaks in summer were consistently noted. Helmet compliance remained low: 19% in Barcelona, 34% in London, and similarly low in the UK Cambridge series [1-3]. Even more concerning, Dela Cruz et al. reported that over 40% of riders exceeded the 15.5 mph legal speed limit, while 7% were documented as intoxicated [2].

Injury mechanisms are typically falls rather than collisions. Falls accounted for 69-86% of incidents in major series, followed by collisions with vehicles or stationary objects [3]. Environmental and behavioral factors contribute: many incidents occur during evening hours, on roads or pavements, and frequently involve riders exceeding legal speed limits or riding without helmets [5,7]. Intoxication with alcohol or recreational drugs has also been noted in 7-36% of cases [9,10].

Helmet use remains alarmingly low globally, though with striking regional disparities. For instance, compliance is exceptionally low in US cohorts, consistently reported below 6%, compared to European series, which report rates of ~20-34% [1,2,4,10]. In Barcelona, only 19% of riders wore a helmet [1], while in London 34% did so [1,2]. Studies from the US and Singapore report even lower compliance rates, ranging from 2.9% to 5.6% [9,10]. This reflects a broader underestimation of the risks associated with e-scooter use compared to other motorized two-wheel vehicles.

Seasonal and temporal trends are also evident. Presentations peak during the summer months and in evening periods, reflecting patterns of recreational and commuting use [1]. Importantly, even in regions where private e-scooter use remains illegal (such as the UK at the time of Hourston’s study), case numbers continue to rise, reflecting widespread availability and adoption [3]. The burden in these regions is often linked to the rapid expansion of rental schemes, a trend also documented in the US, Australasia, and across Europe [2,11]. The associated orthopedic surgical and hospitalization costs have been identified as a substantial healthcare burden in multiple regions [7,12].

Mechanism of injury

The understanding of injury mechanisms is crucial for interpreting the specific fracture patterns observed. Most e-scooter incidents are characterized by falls, but beyond the frequency, the mechanics of these falls are critical in shaping the orthopedic injury patterns that follow. The predominant trajectory involves a forward projection of the rider, typically after sudden braking, hitting a curb, or encountering surface irregularities such as tramlines or potholes. This often results in a fall onto an outstretched hand, explaining the high prevalence of distal radius and radial head fractures reported across series [1,2].

Other mechanisms include direct side-impact collisions with vehicles or street furniture, producing lateral loading forces that increase the risk of lower limb fractures, including tibial plateau and femoral neck injuries [2,3]. Some patients sustain injuries while attempting to step off or dismount a moving scooter, leading to awkward torsional forces and ankle or knee injuries [1]. Case descriptions also highlight over-the-handlebar ejections, producing axial loading through the upper extremities and occasional spinal or rib fractures [2,7].

Intoxication and speed further modify the mechanism. Alcohol or drug use impairs protective reflexes, increasing the likelihood of unprotected head-first impact [2], while excessive speed amplifies energy transfer during collisions, raising the risk of open or periarticular fractures [5,9].

Finally, pedestrian-involved mechanisms, either being struck by a scooter or tripping over parked devices, represent a small but important subset, adding complexity to the injury spectrum [7].

Orthopedic injury patterns

Anatomical Distribution

Upper limb fractures predominate in nearly all cohorts. In Barcelona, 56-63% of fractures involved the upper limb, with distal radius and radial head fractures most frequent [1]. The London multicenter study reported 56% upper limb and 41.9% lower limb fractures [2]. Similar distributions have been described in German and Australasian cohorts [11,13,14]. Forward falls onto outstretched hands are the presumed mechanism, consistent with the high rate of wrist and elbow injuries.

Lower Limb and Axial Injuries

Lower-limb injuries, while less common, are often more severe. These include ankle, tibial plateau, and femoral neck fractures, the latter reported even in relatively young adults [2-4]. Axial injuries (spine and rib fractures) are infrequent but recognized [11]. The Australasian experiences also document pelvic and acetabular fractures requiring operative management [13].

Open and Complex Injuries

A significant proportion of fractures are open. In London, 12.9% of fractures were open, including tibial and distal humerus fractures [2]. Hourston et al. also reported open tibial fractures in their UK case series [3]. Similar injury patterns are noted in North America, where Ishmael et al. reported complex periarticular injuries requiring fixation and Puzio et al. described open fractures with degloving wounds [6,15].

Associated Musculoskeletal Injuries

Dislocations (most commonly shoulder), soft tissue lacerations, and crush injuries accompany fractures in a subset of patients [2,3,16]. Head and facial trauma are frequent co-injuries, especially where helmet use is low [4,10,17]. Neurosurgical involvement is required in some cases, highlighting the multisystem nature of these presentations [18].

Injury severity

Operative Rates

In Barcelona, 25% of patients required surgery, with 49.5% of fractures treated operatively [1]. London reported 30% of patients undergoing surgical management [2]. Hourston’s UK case series also highlighted a high operative burden relative to case numbers [3]. Similar findings were documented in the US, where complex orthopedic procedures were required [6].

Complexity and Resource Intensity

Open and periarticular injuries increase theater demand and length of hospital stay. In London, the mean length of stay for admitted patients was 4.9 days [2]. Hourston estimated that operative cases substantially drive NHS costs [3]. Campbell et al. quantified direct surgical costs in New Zealand, showing a significant economic impact [12].

Rare but Severe Outcomes

While most orthopedic series report few ICU admissions or deaths, Kobayashi et al. described multisystem trauma and fatalities [16]. Neurosurgical emergencies, including subdural hematomas, have also been highlighted [18].

Outcomes

Short-Term Outcomes

The majority of patients are discharged from the ED, but a considerable minority require admission and surgery. Reported complications include infection in open fractures, wound healing problems, and postoperative stiffness [3]. Bloom et al. demonstrated the wider community burden of recurrent hospital presentations [19].

Medium- to Long-Term Outcomes

A critical limitation of the current evidence base is the almost complete absence of robust long-term functional outcomes data. While existing reports highlight time off work, prolonged rehabilitation, and functional impairment after periarticular and open fractures [2]. Economic analyses from the UK and abroad confirm a measurable healthcare burden [7,12]. Several authors emphasize the need for prospective outcome registries to capture long-term sequelae [1].

Future Directions

Several authors emphasize that future research must prioritize prospective cohort studies incorporating validated patient-reported outcome measures to evaluate long-term sequelae [1,2]. Establishing outcome registries would enable more precise assessment of not only surgical success but also patient-centered endpoints, such as return to employment, activity level, and psychosocial well-being.

Management strategies

The management of e-scooter-related orthopedic injuries follows established trauma principles, but the high energy and specific patterns of these injuries warrant emphasis on several key areas.

Triage and Initial Assessment

Immediate priorities follow standard trauma principles: airway, breathing, circulation, and neurological status for multisystem patients, and limb-threatening issues for isolated orthopedic injuries [3]. Patients presenting after e-scooter crashes often have extremity fractures, wounds, and head injuries; clinicians should have a low threshold for full trauma evaluation when the mechanism suggests high energy (e.g., vehicle collision and loss of control at speed) [4].

Imaging

Plain radiographs are first-line for suspected fractures of upper and lower limbs; CT should be used to characterize complex periarticular, pelvic, or acetabular fractures and to plan fixation when standard radiographs are inadequate [6]. Head CT is indicated for any altered conscious state, focal neurology, or significant head impact, given the documented rate of head injuries in several series [4]. A low threshold should also be maintained for CT of the wrist and elbow, owing to the high prevalence of complex intra-articular fractures in these regions.

Wound Management and Open Fractures

Open fractures require early intravenous antibiotics, tetanus cover, and timely operative irrigation/debridement per established open-fracture guidelines; several cohort reports describe open tibial and distal humeral injuries that followed this pathway [2]. Early involvement of plastic surgery is advised for complex soft tissue loss or degloving injuries [3].

Operative Decision-Making

Indications for surgery follow standard orthopedic principles (displaced intra-articular fractures, unstable long-bone fractures, open fractures, neurovascular compromise, and displaced periarticular injuries). Reported operative rates from large cohorts (Barcelona ~25% overall; London ~30% of patients) reflect that many e-scooter fractures require fixation rather than conservative care [1,4]. Surgeons should anticipate a subset of complex reconstructions and staged procedures for severely contaminated or comminuted injuries.

Pain, Thromboprophylaxis, and Medical Optimization

Analgesia, splintage, and early physiotherapy mobilization when safe are important for functional recovery [2]. For admitted patients, follow local venous thromboembolism prophylaxis protocols based on mobility, fracture site, and patient risk factors; several multicenter series reported inpatient admissions and typical orthopedic inpatient risks [7].

Follow-Up and Rehabilitation

Periarticular injuries frequently need early supervised physiotherapy to reduce stiffness and optimize function; the authors note medium-term rehabilitation needs but highlight a paucity of standardized follow-up data in the literature [1]. Establish clear outpatient pathways, wound review, radiographic union checks, and physiotherapy referral to reduce secondary complications and readmissions [19].

Multidisciplinary Involvement

Because head, facial, and occasionally thoracic/abdominal injuries can coexist, early liaison with neurosurgery, maxillofacial, and general surgery should be considered where exam/mechanism dictates; several series recorded neurosurgical referrals for intracranial injury [4].

Economic impact

Direct Healthcare Costs

E-scooter injuries impose measurable direct costs through ED attendances, inpatient admissions, operating theater time, imaging, and outpatient follow-up. Regional analyses and single-center cost studies show that operative cases and admissions are the principal drivers of expense [7]. Campbell et al. quantified orthopedic surgery costs in New Zealand following the introduction of rental schemes and reported significant per-case surgical costs [12].

Service Burden

Increased theater demand, clinic appointments, and rehabilitation referrals were reported in urban centers (London, Barcelona, and Auckland), with operative caseloads forming a substantial portion of orthopedic workload after e-scooter introduction or expansion [14]. Mean lengths of stay for admitted patients (e.g., London cohort ~4.9 days) further increase resource use [2].

Population-Level Economic Effects

Broader analyses using regional datasets indicate higher emergency service utilization and cumulative costs as rental schemes scale up; Bekhit et al. modelled regional healthcare costs and concluded e-scooter injuries represent a notable addition to trauma-service budgets [7,17]. Indirect costs (time off work, lost productivity, rehabilitation, and long-term disability) are mentioned across series but remain poorly quantified in a standardized way [7].

Cost-Effectiveness of Prevention

Although formal cost-effectiveness studies are limited, the literature implies that interventions that reduce severe injuries (helmet programs, speed limiting, rider education, and infrastructure improvements) could produce downstream savings by lowering operative and inpatient rates, an argument invoked by several authors calling for preventive policy [12].

Prevention

Helmet Use and Personal Protective Equipment

Low helmet uptake is a consistent finding (Barcelona 19.1%; London 34% in the cited series) and is associated with higher head-injury rates in broader surveillance studies; promoting helmet use (through legislation, rental operator provision, or incentives) is a primary preventive measure suggested by multiple authors [1,4,10]. Dela Cruz et al. found that only one-third of UK riders wore helmets, and over 40% rode above the speed limit [2]. They advocate for technological controls such as geo-fencing to enforce speed restrictions. Coelho et al. reported Spanish regulations mandating helmet, light, and bell use, with fines up to €500 for non-compliance [1]. Hourston et al. warned that UK legalization without protective infrastructure would likely exacerbate the injury burden [3].

Furthermore, comparative studies cited within these papers reveal that the fracture incidence from e-scooter accidents (≈47%) exceeds that of bicycle or motorbike accidents (23-40%) and even motor vehicle trauma (≈37%) [20,21]. This supports the classification of these injuries as high-energy events requiring policy-level intervention. Evidence from La Torre et al. shows helmet laws can reduce head injury risk by up to 44%, supporting legislative enforcement [22].

Regulation of Vehicle Speed and Design

Speed is repeatedly implicated in injury severity; speed-limiting measures (electronic governor settings on rental fleets and local speed caps) are recommended to reduce kinetic energy in crashes, with geofencing technology proposed to enforce slow zones. Several policy-focused reports and the London authors highlight speed control as a modifiable risk factor [5,13].

Rental Operator Controls and Technology

Operator-level interventions, mandatory in-app safety briefings, enforced helmet reminders, photo verification, nightly parking rules, and geofencing to prevent riding in high-risk zones have been implemented in many pilot schemes and are advocated in the literature as practical harm-reduction measures [13]. Evidence for effectiveness is emerging but not yet definitive.

Licensing, Age Limits, and Rider Training

Some studies and policy briefs recommend age restrictions (e.g., minimum 16-18 years), mandatory basic training or online modules, and licensing for high-speed devices; driver-education analogies (moped/scooter training) have been suggested to reduce incidence and severity [23].

Alcohol/Drug Policies and Enforcement

Intoxication at the time of injury is a recurring risk factor in many cohorts; enforcement of drink-riding laws, in-app reminders, and cutoff rental windows overnight could reduce alcohol-related crashes. Several series reported appreciable proportions of incidents associated with intoxication [9,17].

Infrastructure and Urban Planning

Provision of segregated cycle lanes, maintenance of road surfaces (to remove potholes and tramline hazards), and clear legal frameworks for where e-scooters may be ridden (road vs. cycle lane vs. pavement) are cited as important system-level interventions likely to reduce crash rates. Cities reporting early rental schemes emphasize infrastructure mismatch as a contributor to injury [5,14].

Public Health Education and Surveillance

Authors call for rider education campaigns (safe riding, helmet use, and visibility at night) and for improved surveillance (mechanism coding in registries and prospective data collection) so the effectiveness of prevention strategies can be evaluated. Several papers note the absence of standardized, long-term outcome and cost data and recommend registry linkage to monitor trends and policy impact [1,7].

## Conclusions

E-scooters have emerged as a convenient yet potentially hazardous mode of transport, contributing to a rising burden of orthopedic injuries. Fractures, particularly of the upper limb, are common, and a significant proportion require operative intervention, including complex reconstructions. These injuries not only challenge trauma services but also impose substantial economic and functional burdens. Effective management requires adherence to established trauma principles, early multidisciplinary involvement, and careful rehabilitation planning. Prevention strategies, including helmet use, speed regulation, rider education, and infrastructure improvements, are critical to reducing both the incidence and severity of injuries. Given the current gaps in long-term functional and economic outcome data, prospective studies and registries are urgently needed to inform clinical practice and public health policy. By combining robust management strategies with targeted prevention efforts, the impact of e-scooter-related orthopedic trauma can be mitigated, improving safety for riders and reducing pressure on healthcare systems.
